# Effects of ecologically relevant acclimation temperature on upper thermal limits of juvenile Chinook salmon (*Oncorhynchus tshawytscha*)

**DOI:** 10.1093/conphys/coag043

**Published:** 2026-07-04

**Authors:** Natalie A Butler, Scott G Hinch, Andrew Lotto, Emma L Lunzmann-Cooke, Kim Birnie-Gauvin, Erika J Eliason

**Affiliations:** Pacific Salmon Ecology and Conservation Laboratory, Department of Forest and Conservation Sciences, University of British Columbia, 2424 Main Mall, Vancouver, BC V6T 1Z4, Canada; Pacific Salmon Ecology and Conservation Laboratory, Department of Forest and Conservation Sciences, University of British Columbia, 2424 Main Mall, Vancouver, BC V6T 1Z4, Canada; Pacific Salmon Ecology and Conservation Laboratory, Department of Forest and Conservation Sciences, University of British Columbia, 2424 Main Mall, Vancouver, BC V6T 1Z4, Canada; Department of Ocean Sciences, Memorial University of Newfoundland, NL A1C 5S7, Canada; Section for Freshwater Fisheries and Ecology, National Institute of Aquatic Resources, Technical University of Denmark, Vejlsøvej 39, Silkeborg 8600, Denmark; Department of Ecology, Evolution, and Marine Biology, University of California Santa Barbara, UCEN Rd Building 535, Goleta, CA 93117, USA; Pacific Science Enterprise Centre, Fisheries and Oceans Canada, 4160 Marine Dr, West Vancouver, BC V7V 1N6, Canada; Department of Ecology, Evolution, and Marine Biology, University of California Santa Barbara, UCEN Rd Building 535, Goleta, CA 93117, USA

**Keywords:** Climate change, critical thermal maximum, plasticity, swimming, thermal tolerance

## Abstract

Pacific salmon currently experience temperatures in freshwater that can reach or exceed their upper thermal limits, but little is known about these limits for early life stages. As such, this study aimed to (i) evaluate the role of thermal acclimation on the upper thermal tolerance of stream-type juvenile Chinook salmon (*Oncorhynchus tshawytscha*) fry and parr and (ii) determine how different methods for assessing thermal limits affect the measured outcome. Both parr and fry life stages were acclimated for 2 weeks at temperatures spanning present (15, 18 and 20°C) and expected future summer levels (24°C). Using fish from each acclimation temperature, we determined the critical thermal maximum at rest (CT_**max**_) and during swimming (CT_**swim;**_ temperature at which fish fatigue from swimming) for both life stages, and further assessed prolonged swim performance and post-swim mortality. CT_**swim**_ was a more sensitive indicator of upper thermal limits compared to CT_**max**_, for both life stages. Fish acclimated to higher temperatures generally exhibited higher thermal tolerance though acclimation capacity was diminished between 20 and 24°C. There was high post-swim mortality for the 24°C acclimation groups in both parr and fry, indicating an upper thermal limit where recovery was impaired. Overall, fry had higher upper thermal tolerance (shown by CT_**swim**_, prolonged swim completion and post-swim mortality rates) compared to parr, demonstrating that life stage thermal tolerance corresponded with seasonal temperature exposure differences. Warm water temperatures exceeding 20°C are now routinely occurring in the study population region and many salmonid habitats worldwide, so we expect increasing mortality rates of early life stages in coming years.

## Introduction

As the climate continues to change, rising water temperatures are becoming a major concern for fish globally, particularly those in freshwater. Temperature is considered ‘the master variable’ (Brett, 1971) due to its influence on dissolved oxygen (DO) and other gases, and because of the direct effect it has on physiological processes given that fish are ectothermic. Furthermore, water temperature can be thought of as an ecological resource in which fish behave to attain and retain thermally preferable environments, similarly to the way they may select feeding sites (Magnussen *et al.*, 1979). As such, thermal tolerance and thermal niche choice become important determinants of growth and survival for fishes. Fish can maintain performance in the face of warming temperatures through neuroendocrine, hormonal and molecular responses; however, if they cannot physiologically compensate, whole-animal reductions in growth, condition or reproduction can manifest (Barton, 2002). Elevated temperatures reduce food conversion efficiency and lead to reductions in growth (Brett *et al.*, 1982; Wendelaar Bonga, 1997). Reduced energy stores and growth may impact a fish’s ability to compete for resources and recover from exhaustive activities, such as avoiding predation and migrating. When thermal limits are reached or surpassed, maladaptive coping mechanisms can lead to death (Poletto *et al.*, 2017).

Upper thermal limits vary among fish populations, species and life stages (reviewed for Pacific salmon in Mayer *et al.*, 2023). Identifying which life stage is most sensitive to warming, or life stage ‘thermal bottlenecks’, is critical for management and conservation. Previous research has identified that early life stages and spawning adults are most sensitive to warming (Dahlke *et al.*, 2020; Madeira *et al.*, 2020), while others have found that adults are most thermally sensitive (Cowan *et al.*, 2023) or mixed results (Turko *et al.*, 2020*;*  Mayer *et al.*, 2023). Furthermore, individuals can exhibit considerable plasticity in upper thermal tolerance arising from acclimation or intrinsic plasticity (Becker and Genoway, 1979; Beitinger *et al.*, 2000; Quigley and Hinch, 2006; Poletto *et al.*, 2017). Plasticity can be considered bounded by a thermal ceiling, established as the highest temperature the fish can endure, and past that point organism-level physiological collapse occurs (Becker and Genoway, 1979; Farrell, 2002; Galbreath *et al.*, 2004). Acclimation processes leading to physiological alterations (e.g. synthesis of heat shock proteins) and morphological changes (e.g. changes to ventricle size and composition, capillary to fibre ratios) allow fish to cope with novel thermal conditions. Thermal acclimation capacity is expected to vary across the life cycle, yet this remains largely understudied.

Numerous methodologies have been used to assess thermal tolerance, and thermal limits derived from different methods can generate different outcomes and perspectives on thermal tolerance (Mayer *et al.*, 2023). Critical thermal maximum (CT_max_) and minimum (CT_min_) approaches use a continual and consistent change in temperature from an acclimation temperature until loss of equilibrium (LOE) is achieved (Cowles and Bogert, 1944). Though the test is not lethal, CT temperatures can be considered functionally lethal temperatures for a fish because they would be unable to perform basic functions (swim, escape a predator, etc.). CT_max_ values must be interpreted cautiously because the rapid warming rate (typically 0.3°C/min) surpasses natural conditions; thus CT_max_ values greatly exceed functional thermal limits. In addition, methodologies exploring more functional limits may not be correlated with the same physiological metrics as these more popular methodologies (Bartlett *et al.*, 2022). While the ecological relevance of this approach has been questioned (for review, see Desforges *et al.*, 2023), and the physiological basis for LOE remains unknown (for review, see Ern *et al.*, 2023) CT_max_ and CT_min_ have been successfully used to compare relative thermal tolerance across life stages, populations and species and to assess acclimation capacity (McKenzie *et al.*, 2020). Beyond lethal limits, managers and conservation biologists often seek sublethal thermal limits to identify the temperatures when normal fish performance (e.g. migration, digestion, growth and reproduction) becomes impaired. Four studies have adapted the CT_max_ protocol to work with swimming fish (CT_swim_) to investigate the physiological mechanisms determining the collapse in performance during warming (Steinhausen *et al.*, 2008; Blasco *et al.*, 2020; Nelson *et al.*, 2021; Ekström *et al.*, 2023). This more ecologically realistic approach uses a fixed aerobic swimming speed while slowly increasing the water temperature until the fish reaches fatigue and quits swimming (Steinhausen *et al.*, 2008; Blasco *et al.*, 2020). While this CT_swim_ method is more ecologically relevant compared to CT_max_ (fish are swimming, warming rate is slower), the rate of temperature increase is still faster than fish would typically encounter in the wild and thus CT_swim_ values must be interpreted within this context. Numerous other performance metrics can be used to assess sublethal thermal thresholds, for example swimming performance (McDonnell and Chapman, 2016; Eliason *et al.*, 2017), aerobic scope (Eliason *et al.*, 2011) and cardiac performance (Gilbert *et al.*, 2024). Because of the myriad of metrics available and the varying information they convey, investigating upper thermal limits through multiple lenses is important to be able to get a more accurate picture of thermal tolerance (Speers-Roesch and Norin, 2016; Mayer *et al.*, 2023). Appropriate management recommendations hinge on understanding how methods compare and which method is most appropriate for a given life stage.

In British Columbia, riverine peak summer temperatures have been increasing (Morrison *et al.*, 2002), and water temperatures in the Fraser River, Canada’s fourth largest river, have increased ~1°C from 1950 to 2015 (Islam *et al.*, 2019), translating into a doubling of the number of summer days where the water temperature exceeded 20°C. Such consequences mean more days where water is reaching the upper temperature limit of many salmonids (Richter and Kolmes, 2005; Mayer *et al.*, 2023). The current and projected warming of rivers in the Pacific Northwest region raises large concerns for the sustainability of Pacific salmon populations. Muñoz *et al*. (2015) predicted that current warming scenarios from the Intergovernmental Panel on Climate Change could result in a 17% chance of catastrophic loss of Chinook salmon (*Oncorhynchus tshawytscha*) by 2100, or 98% in a worst-case-scenario warming (Christensen *et al.*, 2013). Chinook salmon are among the most widely studied Pacific salmon species in terms of thermal tolerance yet only a small number of studies have examined the juvenile life stage, and few examined thermal tolerance with ecologically relevant methods (Zillig *et al.*, 2021; Mayer *et al.*, 2023). Juvenile life stages are important to study because the freshwater rearing habitat can be dynamic, ephemeral and susceptible to anthropogenic change (Zillig *et al.*, 2021). Pacific salmon with longer fresh water residency times (e.g. stream-type Chinook salmon reside in freshwater for 1 or more years) may be more susceptible to these challenges than those who have more immediate out migrations (e.g. pink and chum salmon). In British Columbia, Chinook salmon typically spawn from late summer to early fall, eggs incubate over the winter and then hatch in early to late spring. Following emergence, juveniles rear in fresh water for 1 or more years (stream-type) or migrate to the ocean after a few months in freshwater (ocean-type). Different life stages will experience different thermal regimes during their freshwater residency. For example, for stream-type juveniles, small fry emerge in late spring encountering warming temperatures throughout the summer while larger parr encounter cooling temperatures throughout late fall and winter months (Quinn, 2018). It is currently unknown how thermal tolerance varies across these freshwater life stages in Chinook salmon.

The objectives of this study were to (i) investigate the role of acclimation temperature spanning the current wild temperature range on the upper thermal tolerance of stream-type Chinook salmon fry and parr from an interior British Columbia population, (ii) assess multiple approaches of thermal tolerance measurement through comparison of progressively more ecologically relevant metrics (CT_max_ vs CT_swim_ vs post-exercise mortality). We hypothesized that (i) fry and parr will differ in their upper thermal limits based on their life stage-specific differences in thermal exposure in the wild (fry thermal tolerance > parr thermal tolerance), (ii) increasing acclimation temperature will allow for an increased CT_max_ and CT_swim_, with CT_swim_ being lower than CT_max_ and (iii) higher acclimation temperatures will elicit increased latent mortality following prolonged exercise due to impaired recovery.

## Materials and Methods

### Experimental animals

Chinook salmon have two distinct life-history types: ocean-type and stream-type. Ocean-type have a freshwater residence time of just a few months before leaving for the ocean, while stream-type will spend at least 1 year rearing in freshwater streams. Numerous Fraser River Chinook salmon conservation units (i.e. meta-populations) have been designated by the Committee on the Status of Endangered Wildlife in Canada as endangered, with the majority of these being stream-type. The current study used stream-type Chinook salmon from Spius Creek Hatchery, British Columbia, Canada.

Each fish was used once for any experiment, CT_max,_ CT_swim_ or the prolonged swim test. All protocols were approved by the Animal Care Committee at the University of British Columbia with the Canadian Council for Animal Care, under animal use protocol (experiment #A19–0284, holding #A19–0056).

### Transport, holding and acclimation

Chinook salmon parr (*N* = 610; 2019 brood year; 6.88 ± 2.42 g; 87 ± 7.5 mm) were collected 15 October 2020 and fry (*N* = 1288; 2021 brood year; 0.71 ± 0.22 g; 41 ± 4.3 mm) were collected 1 May 2021 from the Spius Creek Hatchery (Nicola River, British Columbia, Canada) and driven 3.5 h to the Pacific Salmon Conservation and Ecology Laboratory at the University of British Columbia (UBC). Parr were transported in 1500 l of freshwater maintained at 10°C with dissolved oxygen greater than 85% the entire trip. Fry were distributed among four 60-l coolers maintained at similar temperature and DO levels to parr.

Upon arrival at UBC, four 375-l recirculating acclimation troughs received 125 parr or 200 fry each, at random (one trough per acclimation temperature). The troughs were partially covered with insulating foam to create shaded areas and fluorescent lighting was set to a naturalistic photoperiod, adjusting the diurnal cycles every 2 weeks with the change in sunset. Temperature was monitored with an Inkbird ITC-306 temperature controller and maintained with 800 W titanium (Aquatop model TH-800) and 300 W titanium (Hydor THEO HY-T11402) heaters. A perforated hose was run through each trough to ensure proper aeration and to avoid stratification. A water pump (Tunze Silence 1073.008) recirculated water. Temperature gradients in each trough were monitored with a Veegee alcohol thermometer at the outflow and a tidbit V2 temperature data logger at the midway. DO was maintained above 75% saturation at outflow and greater than 80% at inflow, measured with an Oxyguard Handy Polaris or a YSI Pro. Water flow was monitored using a Gardena Digital flow meter, and maintained between 0.5 and 1.7 l/min. Oxygen and temperature gradients, nitrites, nitrates and ammonia were checked twice per day Monday–Friday, and once per day Saturday and Sunday. Nitrites, nitrates and ammonia were all below the minimum detection range throughout the study. Fish were fed four times a day Monday–Friday, and once or twice a day Saturday/Sunday with EWOS Pacific Plus complete feed for salmonids (1.5 mm) for parr and Skretting Bio-Vita (mix of sizes #0 and #1) for fry. Fish acclimated to 15, 18, 20 and 24°C were fed 1.5, 2.0, 2.5 and 3.5% of their body mass, respectively (Diana, 1995).

All acclimation troughs began at 15°C and temperature was slowly increased to the target acclimation temperatures of 15, 18, 20 and 24°C over 3 days (increase of 3°C per day until target temperature was reached). Treatment temperatures were measured from temperature loggers placed midway of each trough (HOBO Tidbit V2 temperature logger). Once the fish reached their acclimation temperature, temperatures were maintained close to target acclimation temperature for 2 weeks before experiments began (mean ± SD, median and mode provided in Table S1). During the experimental period, fish were maintained at their acclimation temperatures, though there were a few isolated instances when the temperature decreased below the target temperature for 1–4 days due to decreases in the ambient city water, after the initial acclimation period and after the CT_max_ trials. These temperature decreases occurred mainly during the 2020 trials over the winter (Table 1). Two mass mortality events in 24°C acclimated parr after 26 and 27 days in the acclimation tank required the acclimation of an additional 50 fish in order to complete the swim performance and CT_swim_ trials. Thus, the 24°C acclimated parr were acclimated for a shorter duration compared to the other groups (Table 1).

**Table 1:** Timeline of experimental events for both fry and parr life stages

**Experimental events**	**Parr (2020)**	**Experimental day**	**Fry (2021)**	**Experimental day**
Acclimation temperature increase	October 21–23	–2 to 0	May 5–7	–2 to 0
Acclimation holding period	October 24–November 7	1 to 14	May 8–22	1 to 14
CT_max_ trials	November 8–9	15 to 16	May 25–28	17 to 20
*Addition of new fish for acclimation due to mass mortality event (24°C)*	*November 20–23*	–*2 to 0*		
*New 24°C Acclimation holding period*	*November 24–December 7*	*1 to 14*		
Swim performance trials	November 15–26 *December 7–8*	22 to 33 *14 to 15*	May 31–June 16	23 to 39
CT_swim_ trials	December 1–3, *11*	38 to 40 *18*	June 21–24	44 to 47

Italics denote a secondary timeline of acclimation required due to mortality during the holding period in 2020.

### Critical thermal maximum

One critical thermal maximum (CT_max_) trial was run for each acclimation treatment and each life stage. For each trial, 15 parr from one acclimation treatment were placed in a 40-l cooler with four heaters affixed to the bottom, four water pumps and two air stones. Pump intakes were covered with filter foam to prevent fish from damage. Fifteen fry from one acclimation treatment were placed in a 17-l cooler with two heaters affixed to the bottom and the same pump configuration as the parr. The starting temperature matched that of the acclimation treatment and fish were allowed to adjust for 30 min in the bath before the trial began. After the adjustment period, water was heated at 0.3°C/min and monitored with a YSI temperature probe. CT_max_ was determined as the temperature when the fish was no longer able to right itself (LOE). Upon LOE (defined as the moment the fish flipped, ventral side up), the individual was netted and then euthanized with buffered tricaine methanosulfonate (MS-222, 250 mg/l). Temperature, length and mass were recorded from each fish. This process was repeated for all acclimation treatments over the course of 2 days; all trials were conducted between 11:00 and 15:00 PST/PDT.

### Swim fatigue temperature

To obtain a more ecologically realistic estimate of upper thermal limits, we performed a swim fatigue temperature (CT_swim_) test, where a group of fish was swum at a fixed aerobic swimming speed while slowly increasing the water temperature until the fish fatigued and quit swimming (Steinhausen *et al.*, 2008; Blasco *et al.*, 2020, Nelson  *et al.*, 2021). One CT_swim_ trial was run for each acclimation treatment and each life stage. A total of 30 fish from a given acclimation treatment and life stage were placed in a flow-through swim flume system with a 45 l swim space and total volume of roughly 300 l (3200 cm long × 15 cm wide × 25 cm high) (Eliason *et al.*, 2017). The water temperature within the flume was initially at their acclimation temperature, and fish were left to adjust to the flume for 30 min at a swimming speed of 3 cm/s for fry and 8 cm/s for parr. Water velocity was monitored using a Swoffler model 2100 series.

The speed was increased every 5 min by 3 cm/s for fry and 8 cm/s for parr until the target swimming speed of 12 and 32 cm/s was reached, respectively. Preliminary tests identified that these measurements ensured the fish were swimming steadily and aerobically against the flow, without any burst and coast swimming activity. Once at the target swimming speed, the water temperature was increased by 1°C every 15 min until the water reached 20°C, then the rate of warming decreased to 1°C every 30 min, using a 300 W heater (Aquatop TH-C500), a 1300 W heater (Pong-Dang heater AJT13K) and a chiller (EcoPlus commercial chiller HGC728707). Fish were considered to have fatigued, and thus reached CT_swim_, after 15 s of continuous body contact with the back grate of the flume and if they did not swim off the grate when prodded once with a net. If the fish swam off the grate and continued to swim during removal, it was allowed to continue swimming. Once a fish reached CT_swim_, it was removed from the trial by netting and the time and temperature were recorded. To minimize disturbance when removing fish, the flume was outfitted with bright lights closest to either extremity of the flume and a black sheet of plastic shading the top of the flume as well as the far side to encourage fish to swim in the middle of the flume, ensuring that those that drifted back were experiencing fatigue. The fish were euthanized by concussion and decapitation, measured for mass and length, and tissues were collected for analysis (data not included here). This process was repeated for each acclimation temperature treatment over 4 days.

### Swimming performance

A standard critical swimming test (*U*_crit_) where fish are swum at increasing velocities until they reach fatigue could not be employed here because of limitations with the swim flume apparatus (flume could only achieve a maximum water velocity of 30 cm/s for fry and 56 cm/s for parr). Thus, we employed a prolonged ramping protocol designed to induce fish to swim for several hours first aerobically (steady swimming) and then anaerobically (burst and coast swimming) near the end of the trial. We expected fish acclimated and tested at optimal swimming temperatures to be able to complete the entire swim protocol while those acclimated or tested at supraoptimal temperatures would not be able to complete the test.

Groups of 25 fish per acclimation temperature and life stage underwent a prolonged group swimming test at every acclimation temperature using the same swim flume as the CT_swim_ test (described above, see also Eliason *et al.*, 2017). To ensure that fish were swimming in the middle of the flume where flow was most laminar, light was shown at both extremities of the swim space, and a black garbage bag was clamped to the top and far side of the flume to create a more desirable swimming area in the middle. Flume temperature was continuously maintained at the target test temperature (i.e. 15, 18, 20 or 24°C) ± 0.8°C for the entire duration of the swim. Fish were placed in the flume at a given test temperature (‘flume temperature’) and allowed to adjust to the flume, swimming steadily at 8 cm/s (parr) or 6 cm/s (fry) for 30 min before the trial began. After 30 min, water velocity was increased every 30 min by 3 cm/s for fry and 8 cm/s for parr, 16, 24, 32, 40, 48 and 56 cm/s over 3 h for parr or 9, 12, 15, 18, 21, 24, 27 and 30 cm/s over 4 h for fry. At these higher velocities, fish were starting to transition to anaerobic, burst and coast swimming. Fish were removed when they fatigued, using the same criteria as the CT_swim_ trials. At removal, time and speed were recorded, the individual was briefly anaesthetized to measure length and mass. After reaching 56 cm/s (parr) or 30 cm/s (fry), the swim trial was terminated and all fish that were still swimming were counted, removed and briefly anaesthetized to measure fork length and mass. Regardless of swim trial completion, fish were then placed in a mesh bucket with air stones in their initial acclimation trough and monitored for recovery after 24 and 48 h, during which time any mortalities were recorded. After 48 h, the fish were released into a sectioned-off end of their acclimation trough, isolated from any fish still naïve to the flume. These fish were then euthanized at the end of the study using buffered MS-222 at 250 mg/l.

### Acclimation response ratio

The acclimation response ratio (ARR) is defined here as a degree change in CT_max_ (or CT_swim_) per degree change in acclimation temperature (equation (1)) (Claussen, 1977). Using ARR is a way of quantifying the ratio between the upper thermal limit and the level of acclimation. For example, an ARR of 1 means that for every degree change in acclimation temperature, there is a one-degree change in CT_max._ This metric is helpful in gauging how ectotherms may respond to changes in the environment, and in some cases, indicate the potential for long-term adaptation to changing temperatures (Morley *et al.*, 2019).


(1) \begin{equation*} \mathrm{ARR}=\frac{\Delta \mathrm{CTmax}}{\Delta T} \end{equation*}

### Statistical analysis

All data were analysed with R 4.2.1 (2021) and RStudio IDE version 2023.03.0+386, and a significance level of *α* = 0.05 was applied for all statistical tests. All measures are given as mean ± SD unless otherwise noted. Spearman rank correlation was used to identify correlations among mass, fork length and condition factor. For fry, mass and fork length were highly correlated (*r* = 0.88), and for parr, mass was highly correlated with both fork length (*r* = 0.90) and condition factor (*r* = 0.67). Mass was chosen as a covariate for modelling to avoid overfitting. Outliers were assessed using Cook’s distance and visual observation on a quantile–quantile plot, and no datapoints were removed (Cook’s *D* < 0.5).

Analysis of variance (ANOVA) was performed to assess the influence of acclimation temperature on mass, fork length and condition factor of each treatment group at the end of the 2-week acclimation period, as well as a two-way ANOVA to assess the difference in these metrics between test types, with acclimation temperature as an independent variable. For both fry and parr, general linear models with a normal distribution were used with CT_max_ and CT_swim_ as the response variable, mass and acclimation temperature as predictor variables. Finally, Bayesian information criterion (BIC) and Log likelihood were used in model selection (Table S2, Table S3). Log likelihood ratio tests showed no significant preference between the nested and full models, and BIC favoured the models containing only acclimation temperature as a predictor variable for both CT_swim_ and CT_max,_ for both fry and parr. *Post hoc* Tukey tests were carried out to assess significant differences.

A two-way ANOVA was performed to compare upper thermal limits by methodology (CT_max_ or CT_swim_) and life stage with measured upper thermal limit as the response variable and test type (CT_max_ and CT_swim_) and life stage (fry and parr) as the predictors.

Due to the low number of observations per swim group (<5 survivors or mortalities per acclimation/flume temperature combination), a pairwise Fisher’s exact test with a Bonferroni *P-*adjustment was used to compare post-exercise mortalities within acclimation groups.

## Results

### Acclimation mortality and body metrics

For fry, there was a short phase of mortality during the acclimation period across all temperature treatments as fish began exogenous feeding which then tapered off; however, higher mortality was sustained in the 24°C-acclimation group (3.5% for 15°C, 1% for 18°C, 1% for 20°C and 23% for 24°C) throughout the study. For parr, no mortality was observed during acclimation until 13 days into the acclimation period, and low levels of mortality (0.91–10%) in the 24°C occurred until two large mortality events happened 26 and 27 days into the study period, where 27 (71%) and 36 (100%) parr died, respectively.

There were no significant differences in mass and fork length among acclimation treatments after 2 weeks of acclimation (i.e. fish tested for CT_max_, Table 2), and condition factor did not yield any significant pairings *post hoc* (summary mean ± SD, Table 2) for parr (Table S4) (mass: *F*_(3,56)_ = 0.576, *P* = 0.633; FL: *F*_(3,56)_ = 0.597, *P* = 0.619; CF: *F*_(3,56)_ = 3.027, *P* = 0.0369). For fry, only condition factor was statistically significant between the 15 and 20°C acclimation groups (mass: *F*_(3,55)_ = 1.76, *P* = 0.165; FL: *F*_(3,55)_ = 0.232, *P* = 0.874; CF: *F*_(3,55)_ = 3.061, *P* = 0.0356, Tukey’s HSD *P* = 0.021). After prolonged exposure to acclimation temperatures (i.e. >6 weeks for the CT_swim_ tests), the 24°C-acclimated fry were smaller than the other groups (Table 2 and Table S5). Minimal differences in mass, fork length or condition factor were detected in parr across tests and acclimation groups (Table 2 and Table S5), though 24°C fish were excluded from the analysis because of the shorter acclimation period.

**Table 2:** Summary mean ± SD of mass (g), fork length (mm) and condition factor of fry and parr at the end of the 2-week acclimation period, during the CT_swim_ trials and during the prolonged swim trials

**Life stage**	**Experimental event (days of acclimation)**	**Acclimation temperature (°C)**	**Mean mass (g) ± SD**	**Mean fork length (mm) ± SD**	**Condition factor ± SD**
Fry	CT_max_ (17–20 days)	15 (*n* = 14)	0.63 ± 0.05	39.36 ± 1.50	0.1034 ± 0.0075
		18 (*n* = 15)	0.68 ± 0.08	38.87 ± 1.73	0.1149 ± 0.0081
		20 (*n* = 15)	0.70 ± 0.10	38.60 ± 3.35	0.1238 ± 0.021
		24 (*n* = 15)	0.64 ± 0.12	38.93 ± 2.76	0.1118 ± 0.028
	Prolonged swim performance (23–39 days)	15 (*n* = 81)	0.68 ± 0.13	41.07 ± 2.14	0.0966 ± 0.0073
		18 (*n* = 80)	0.67 ± 0.17	40.84 ± 3.10	0.0971 ± 0.0085
		20 (*n* = 81)	0.70 ± 0.18	40.44 ± 3.14	0.1036 ± 0.0012
		24 (*n* = 80)	0.68 ± 0.16	39.41 ± 2.44	0.1086 ± 0.015
	CT_swim_ (44–47 days)	15 (*n* = 30)	0.71 ± 0.17	42.03 ± 3.25	0.0951 ± 0.010
		18 (*n* = 30)	0.81 ± 0.18	43.93 ± 3.34	0.0949 ± 0.0072
		20 (*n* = 30)	0.93 ± 0.24	45.8 ± 3.98	0.0940 ± 0.0064
		24 (*n* = 31)	0.46 ± 0.15	37.4 ± 2.76	0.0858 ± 0.011
Parr	CT_max_ (15–16 days)	15 (*n* = 15)	7.12 ± 2.36	85.5 ± 8.3	0.1098 ± 0.011
		18 (*n* = 15)	6.98 ± 1.51	86.5 ± 3.6	0.1070 ± 0.016
		20 (*n* = 15)	6.89 ± 1.81	88.3 ± 7.0	0.0988 ± 0.010
		24 (*n* = 15)	6.26 ± 1.97	85.3 ± 8.3	0.0994 ± 0.011
	Prolonged swim performance (22–33 days for 15, 18 and 20°C; 14–15 days for 24°C)	15 (*n* = 101)	8.05 ± 3.05	88.23 ± 9.16	0.1124 ± 0.016
		18 (*n* = 104)	8.22 ± 10.77	85.80 ± 8.14	0.1236 ± 0.11
		20 (*n* = 100)	6.60 ± 2.14	84.41 ± 6.94	0.1068 ± 0.013
		24 (*n* = 95)	6.21 ± 2.17	84.63 ± 10.01	0.1456 ± 0.47
	CT_swim_ (38–40 days for 15, 18 and 20°C; 11 days for 24°C)	15 (*n* = 29)	7.58 ± 2.79	88.83 ± 8.34	0.1038 ± 0.016
		18 (*n* = 32)	7.08 ± 3.24	86.88 ± 8.86	0.1021 ± 0.003
		20 (*n* = 31)	6.08 ± 1.66	86.00 ± 5.98	0.0940 ± 0.001
		24 (*n* = 9)	7.09 ± 2.70	88.89 ± 8.87	0.0976 ± 0.003

### CT_max_ and CT_swim_

**Figure 1:**
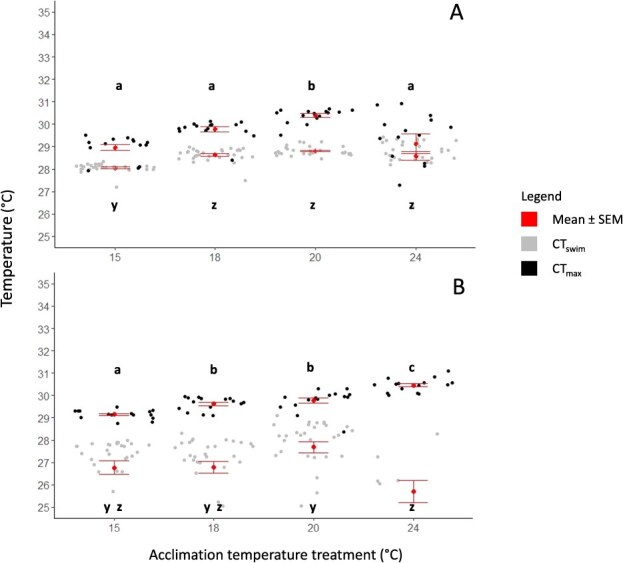
Mean ± SEM of upper thermal limits of fry (**A**) and parr (**B**) based on CT_max_ (black) and CT_swim_ (grey) protocols. Dot and whiskers denote mean and SEM. Different letters denote significant differences within a protocol (a–c denote parr significance, x–z fry). Within each life stage, CT_max_ was always higher than CT_swim_ for each acclimation temperature (ANOVA; fry*: F*_(1,174)_ = 86.75, *P* < 0.001; parr: *F*_(1,157)_ = 171.071, *P* < 0.001)

CT_max_ did not differ between fry and parr *F*_(1, 114)_ *= 1.590, P* = 0.21) (Fig. 1). However, parr had a significantly lower CT_swim_ (by ~1.78°C on average across all acclimation groups) than fry (*F*_(1, 217)_ = 100.495, *P* < 0.001). CT_max_ was significantly higher than CT_swim_ in both fry and parr (by 0.5–1.5°C in fry; *F*_(1,174)_ = 86.75, *P* < 0.001 and by 2.1–4.8°C in parr; *F*_(1,157)_ = 171.071, *P* < 0.001).

Acclimation temperature significantly affected both CT_max_ (fry: *F*_3,55_ = 7.416, *P* = 0.00294; parr: *F*_3,56_ = 38.47, *P* < 0.0001) and CT_swim_ (fry: *F*_3,116_ = 9.8, *P* < 0.0001; parr: *F*_3,98_ = 4.505, *P* = 0.00527), with upper thermal limits increasing with acclimation temperature until 20°C, followed by a plateau or decline between 20 and 24°C. Correspondingly, the ARR (Table 3) in fry shows an increase in acclimation response in CT_max_ between 15 and 20°C, followed by a loss of acclimation capacity at 24°C. During the CT_swim_, there was a loss of acclimation response between both 18 and 24°C, demonstrated by the plateau (CT_swim_) or decrease (CT_max_) between 20 and 24°C (Fig. 1). Parr acclimation response for CT_swim_ decreased while CT_max_ plateaued between 20 and 24°C (Fig. 1).

**Table 3:** ARR of parr and fry between CT_max_ and CT_swim_ protocols

**∆*T* acclimation (°C)**	**Fry**	**Parr**
CT_max_		
15–18	0.35 (*n* = 29)	0.16 (*n* = 30)
18–20	0.90 (*n* = 30)	0.11 (*n* = 30)
20–24	−0.33 (*n* = 30)	0.16 (*n* = 30)
CT_swim_		
15–18	0.16 (*n* = 60)	0.005 (*n* = 62)
18–20	0.11 (*n* = 60)	0.45 (*n* = 63)
20–24	−0.05 (*n* = 60)	−0.61 (*n* = 40)

### Swim performance and latent mortality

Prolonged exercise performance overall was comparable across acclimation temperatures and life stages. Completion rates were typically between 80 and 100%, with the notable exception of an 8% completion rate for 24°C acclimated parr swim at 24°C (Table 4).

**Table 4:** Percent completion rate of the prolonged swim trial for both fry and parr

**Life stage/acclimation temperature (°C)**	**Flume temperature (°C)**			
	**15**	**18**	**20**	**24**
Fry				
15	100	100	100	100
18	100	100	100	100
20	100	100	100	100
24	80	100	100	90
Parr				
15	100	100	96.2	84
18	100	96	96.6	88
20	88	80	100	96
24	91.3	84	100	8

**Figure 2:**
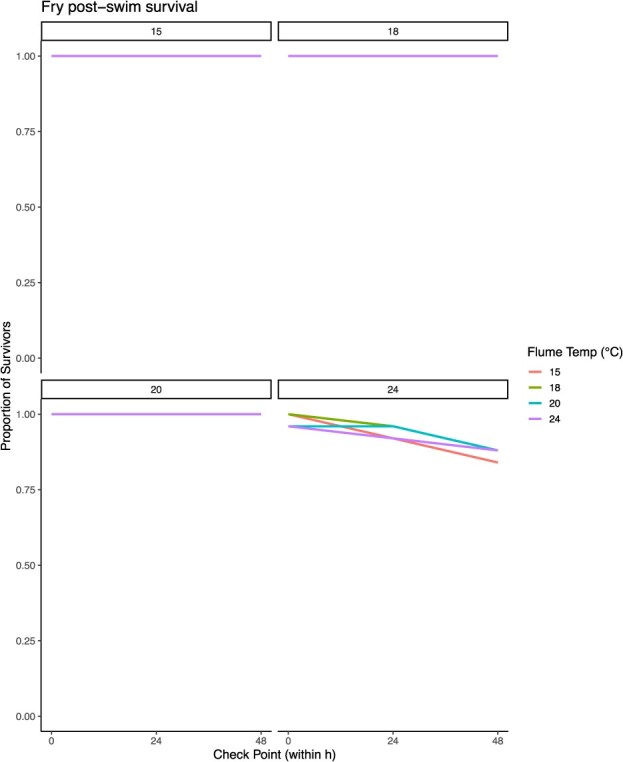
Proportional survival of fry, faceted by acclimation temperature treatment. Proportional survival at each check time is calculated from the mortalities in the pool of remaining fish at that time.

**Figure 3:**
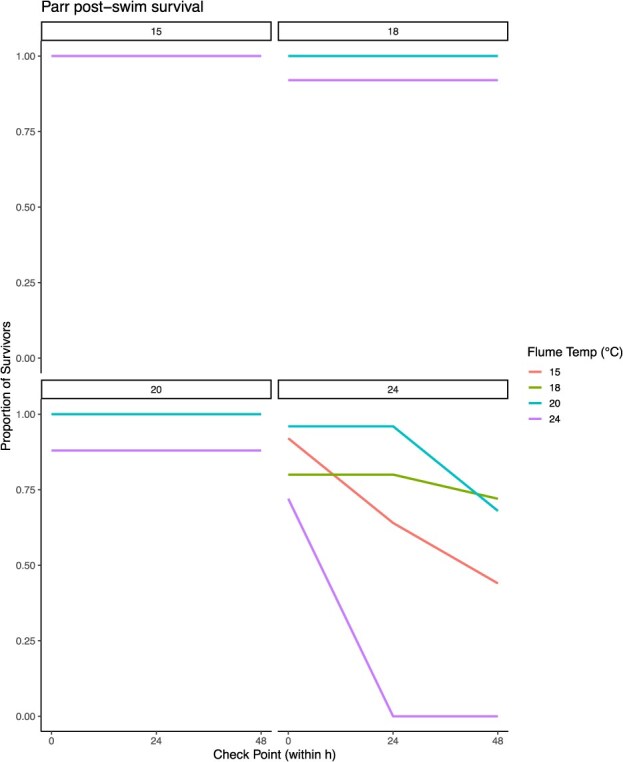
Proportional survival of parr, faceted by acclimation temperature treatment. Proportional survival at each check time is calculated from the mortalities in the pool of remaining fish at that time.

For fry, all post-swim mortality events involved the 24°C-acclimation swim groups (Fig. 2). Similarly, parr mortalities occurred following all swim flume temperatures tests for 24°C-acclimation fish (Fig. 3). In addition, post-swim mortalities also occurred in 18 and 20°C-acclimated groups swum at 24°C in parr (Fig. 3). There were no significant (*P* = 1) differences in mortality between flume groups in the 24°C acclimation treatment group in fry. There were no significant differences in mortality between flume temperature test groups for 18°C-acclimated or 20°C-acclimated parr (*P* > 0.05, Table 5). There were significant differences in mortality in the 24°C acclimation treatment between the 24°C flume treatment and 15°C (*P* = 0.001), 18°C (*P* < 0.001) and 20°C (*P* < 0.001).

**Table 5:** Results of the pairwise fisher test with Bonferroni *P*-adjustment for parr post-swim mortality data

**Acclimation temperature (°C)**	**Flume temperature treatments (°C)**					
	**15/18**	**15/20**	**18/20**	**15/24**	**18/24**	**20/24**
18	*P* = 1	*P* = 1	*P* = 1	*P* = 0.49	*P* = 0.49	*P* = 0.49
20	*P* = 1	*P* = 1	*P* = 1	*P* = 0.235	*P* = 0.235	*P* = 0.235
24	*P* = 0.507	*P* = 0.924	*P* = 1	*P* = 0.001	*P* < 0.001	*P* < 0.001

A 15°C acclimation treatment was excluded due to no mortality. Fraction denotes the flume temperature treatment comparisons (e.g. 15/18 compares fish swum at 15°C vs fish swum at 18°C).

## Discussion

Our first objective was to investigate the role of acclimation temperature on the upper thermal tolerance of Chinook salmon fry and parr. Warm acclimation increased upper thermal tolerance, though both fry and parr reached an upper ceiling for acclimation capacity at 20°C. Overall, fry had higher thermal tolerance compared to parr, as demonstrated by a higher CT_swim_, most fish (>80%) completing the prolonged swim trial at 24°C, and improved survival following the exercise trials. This aligns with differences in seasonal temperature exposure between the two life stages; small fry experience warmer temperatures during late spring/early summer compared to larger fry in late fall/early winter. Below, we discuss how methodology influences the estimate of upper thermal limits, the interpretation of acclimation capacity and life stage comparisons. All told, this study demonstrates the importance of investigating upper thermal limits through multiple lenses to best inform management practices.

Phenotypic plasticity plays a key role allowing fish to respond to changing environmental temperature and expand their upper and lower thermal limits. Given that acclimation processes can take weeks to fully complete, the current study allowed fish to acclimate for ≥2 weeks prior to commencing the performance trials. Mortality did occur in the holding tanks during the acclimation period, particularly for 24°C acclimated parr (100% mortality by 27 days of acclimation). There was a noticeable decline in body condition past the 2-week mark in the 24°C acclimation group for both life stages, which may indicate that metabolic demands driven by the water temperature were not being entirely met despite providing maintenance ration intended to maintain mass after 2 weeks of acclimation. This suggests that 24°C is suboptimal for growth for both fry and parr life stages.

Fulton’s condition factor (Ricker, 1975) helps quantify the effect of chronically elevated temperature on the relationship between length and mass, as this morphometric is considered analogous to energy reserves, and has been shown to be positively correlated with lipid stores in some fish (Herbinger and Friars, 1991; Chellappa *et al.*, 1995; Mozsár *et al.*, 2015). A positive correlation between body condition factor and CT_max_ would suggest a link between body condition and upper thermal tolerance; however, body condition was not a significant predictor of CT_max_ in this study, though other studies have found that high mass and low condition factor was a significant predictor of increased CT_max_ in brown trout (*Salmo trutta*) (Desforges *et al.*, 2021), and Atlantic salmon (*Salmo salar*) in better condition may have a higher thermal tolerance (Bartlett *et al.*, 2022) and survival time in a similar thermal challenge (Robinson *et al.*, 2008). In addition, Turko *et al.* (2020) demonstrate how condition may have a variable effect on thermal tolerance depending on age in redside dace (*Clinostomus elongatus*), highlighting the relationship between body condition and thermal tolerance may not be straightforward.

Acclimation capacity plays a key role in evaluating resiliency when faced with climate change. Acclimation in salmon is found to hinge on several factors: population, historical thermal regimes and temperature exposure (Mayer *et al.*, 2023). Faced with increasingly thermally challenging environments, their resiliency to these environments is paramount for survival. ARR is a helpful metric gauging how ectotherms acclimate to a new thermal environment over a relatively short period of time. Here, both fry and parr clearly demonstrated the capacity to increase their upper thermal tolerance with warm acclimation (ARR ranged from 0.005 to 0.45 between 15–18 and 1820°C for CT_max_ and CT_swim_). However, we observed impaired acclimation capacity in 24°C-acclimated fish, suggesting that both fry and parr have reached a thermal ceiling above 20°C, or were unable to acclimate entirely. Notably, we experienced some difficulty maintaining constant acclimation temperatures in the warmer parr treatments (particularly 20 and 24°C), with intermittent temperature declines during acclimation. This reduction in high temperature exposure during acclimation may therefore underestimate the magnitude of temperature sensitivity in parr. Morley *et al.* (2019) performed an extensive meta-analysis of reported ARR for 319 species, using it as a metric for upper thermal tolerance and estimating thermal safety margins for groups of species with modelled global climate change. Mid-latitude fish (25–55°), such as salmon, had an estimated overall ARR of 0.06. In the present study, the ARR ranged from −0.49 to 0.45, with a mean ARR of 0.05, which is very close to the number reported by Morley *et al.* (2019), corroborating our population’s measured acclimation capacity to the literature. Acclimation capacity is an important metric as populations that inhabit the edge of their thermal range may have limited acclimation capacity as they reside close to their thermal ceiling already, demonstrated by Van Wert *et al.* (2023) in two Chinook salmon populations acclimated to ambient (11–12°C) temperature conditions. Both had similar mortalities at 18°C, but as temperature increased beyond this, those with a shorter and cooler migration had significantly higher mortalities than the population with a much warmer and arduous migration. Additionally, Dressler *et al.* (2023, 2025) found *Oncorhynchus mykiss* populations at their southern range exhibit differing CT_max_ and acclimation capacities based on the thermal regime of their native stream. Warm-adapted fish had a higher overall CT_max_ but less plasticity than the cold-adapted fish.

Our second objective was to compare methodologies for estimating upper thermal limits. By using a common group of fish undergoing a common thermal acclimation protocol, we evaluated how three methods (CT_max_, CT_swim_ and post-exercise survival) compare across life stages and acclimation groups. This study demonstrated that different methodologies influence the estimate of upper thermal limits, life stage comparisons and the assessment of acclimation capacity. Traditional critical thermal methodology (CTM) approaches carry little semblance to the thermal experience in the wild due to the rapid change in temperature compared to wild conditions and the extreme endpoint of LOE, which is not helpful as a standalone metric for management (Mayer *et al.*, 2023). However, it is a common metric that can be easily compared across studies and species (Desforges *et al.*, 2023). The traditional CT_max_ methodology allows the measurement of absolute upper thermal limits, where fish are functionally dead. We found no difference in CT_max_ between fry and parr, though they did differ in their acclimation response. Specifically, we found that parr did not exhibit a reduction in ARR with warming, whereas fry did. If CT_max_ was the only upper thermal tolerance test used, we would simply conclude that upper thermal limits are similar between life stages with fry having reduced thermal acclimation capacity. However, the CT_swim_ methodology helps develop a lower and more realistic thermal threshold, where burst and coast swimming patterns are seen before fatigue, behaviourally indicating the transition from aerobic to anaerobic metabolism. As expected, CT_max_ yielded higher values compared to CT_swim_ (~0.5–1.5°C higher in fry and ~2.1–4.8°C higher in parr). Fry had significantly higher CT_swim_ and improved ARR compared to parr. Specifically, parr acclimated to 24°C demonstrated a dramatic reduction in CT_swim_ and a decrease in ARR at the highest acclimation temperatures. Thus, the CT_swim_ protocol suggests that parr have reduced upper thermal tolerance and inferior acclimation capacity compared to fry. Though the CT_swim_ protocol is more ecologically relevant because of the slower temperature ramp rate and fish are actively swimming, it likely overestimates functional upper thermal limits. Our third method, the prolonged swim, helps infer the effects of acclimation temperature and flume temperature on swim performance and subsequent survival. Most fish successfully completed the prolonged swim trial, with the exception of 24°C acclimated parr swum at 24°C, indicating minimal differences in swim performance across life stage or acclimation experience. This was somewhat surprising because we anticipated that both life stages would have impaired swimming performance at 24°C, given the long duration of the swim trial (several hours). Notably, many studies conclude immediately following a swim test and do not assess recovery and survival. Had we ended the trial after the swim test, we would have erroneously concluded that all fish had equivalent thermal performance, with the exception of 24°C-acclimated parr swum at 24°C. However, there were clear differences in post-exercise mortality. Post-swim mortality occurred in all 24°C-acclimated groups. Notably, even parr acclimated to cooler temperatures (18 and 20°C) but swum at 24°C displayed post-swim mortality. Thus, though both fry and parr can swim several hours at 24°C, it is clear they are unable to recover from prolonged, strenuous swimming (analogous to migrating or actively foraging in high flow areas). It is interesting that cold acclimated fish were able to swim at 24°C with little to no mortality, suggesting that the fish are able to physiologically perform during brief exposures (i.e. hours) at 24°C without notable negative impacts. Collectively, these three methodologies suggest that 24°C exceeds the optimal thermal threshold for both parr and fry. Furthermore, these studies collectively indicate that fry have higher upper thermal limits compared to parr, supporting our hypothesis that fry and parr will differ in their upper thermal limits based on their life stage-specific differences in thermal exposure in the wild (smaller fry encounter warmer temperatures during late spring and summer months while larger parr encounter cooler temperatures during fall and winter).

The physiological mechanisms determining the upper limits likely differ substantially across the three methods tested here. There is considerable debate about the mechanism underlying the LOE that occurs at CT_max_ (for review, see Ern *et al.*, 2023), and it is suggested that these mechanisms can be based on three foundational molecular effects including: reaction rates, protein structure and membrane fluidity, but there is unlikely to be one unifying mechanism for thermal death in fish. For CT_swim_, it is likely that fish cease swimming at high temperatures because of insufficient oxygen delivery to meet the increased demand associated with warming (Steinhausen *et al.*, 2008; Blasco *et al.*, 2020; Ekström *et al.*, 2023). As temperatures warm, oxygen demand increases and the fish switch from steady-state aerobic swimming to burst-and-coast, unsteady anaerobic swimming. This is clear evidence that there is a mismatch between oxygen demand and delivery. Fish can only sustain anaerobic swimming for a finite amount of time before reaching fatigue. It remains unclear why some individual fish can sustain higher temperatures before reaching fatigue compared to others, though it may be related to improved oxygen delivery or superior anaerobic capacity. Finally, researchers have been seeking to understand why fish die following exhaustive exercise for >60 years (Black, 1958; Wood *et al.*, 1983; Holder *et al.*, 2022). The inability for some 24°C-acclimated fish to recover from the prolonged swim test may indicate greater reliance on anaerobic metabolism and thus a greater accumulation of lactate, higher ionic and osmotic imbalance and a greater oxygen debt accrued during the swim trial (Birnie-Gauvin *et al.*, 2023). Temperature and stress can affect digestion and nutrient absorption (Volkoff and Rønnestad, 2020); thus, the fish in the higher acclimation temperature treatments may have had fewer resources and insufficient energy reserves to clear lactate and regain homeostasis following the swim test (Brett *et al.*, 1982; Wendelaar Bonga, 1997), in addition to the added stress of coping with the high temperature acclimation environment. The mortality following the swim may also be the result of cumulative thermal stress, which has been explored in recent studies (Iacarella *et al.*, 2024).

Our study fish came from the Nicola River in southcentral British Columbia, which is characterized in the summer by high temperatures (air temperatures in July and August regularly exceed 30°C) and low precipitation, and has extensive land use activities including agriculture and forestry which are leading to further stream warming in summer months in this river (Ulaski *et al.*, 2023) and reduced primary productivity (Warkentin *et al.*, 2022). In this region, stream temperatures have been recorded exceeding 24°C (Hinch, unpublished data). Nicola River stream-type Chinook Salmon are likely now rearing in summer temperatures that are approaching or exceeding their thermal tolerance, based on our studies results. Our measured thermal tolerance limits exceed those in the published literature for Chinook salmon (see Mayer *et al.*, 2023 for summary) likely due to local adaptation to the aforementioned semi-arid environment. Richter and Kolmes (2005) summarize published lethal temperatures for Chinook salmon, some of the earliest reported being 25.1°C (Brett, 1952) and 24.9°C (Orsi, 1971). Fish were acclimated to 20, 24 and 21.1°C, respectively. It is important to note these metrics are upper incipient lethal temperature (UILT), not CT_max_. These temperatures are below our measured upper thermal tolerance, which is likely a result of both population differences and methodology.

Studies that are used to support fisheries or habitat management decisions should be chosen in a manner that represents the information that is most needed by managers. Tailoring methodologies to make them more ecologically relevant (Desforges *et al.*, 2023) will help approximate more accurate thermal tolerance ranges (Mayer *et al.*, 2023). This research demonstrates the need for managers and practitioners to examine the available literature through a critical lens when making management decisions. Studies that compare thermal tolerance across groups of fish exclusively using CT_max_ are likely to miss important differences, as shown here. Instead, we recommend using multiple, ecologically relevant methods to evaluate thermal tolerance to gain a more complete picture of salmonid thermal tolerance. Furthermore, by using a relatively long experimental thermal acclimation period, we discovered that chronic temperature exposure, close to the published lethal maximum (Richter and Kolmes, 2005), may not lead to death immediately but reduces the ability of fish to withstand and recover from secondary stressors. Furthermore, we identified that fry and parr only have the capacity for thermal plasticity up to 20°C. If these fish experience temperatures beyond 20°C, acclimation cannot improve their thermal tolerance and they must (i) cope (e.g. reduce behaviours, wait out the deleterious temperature); (ii) move (e.g. migrate to more favourable thermal environments, if possible); (iii) adapt (can take 4–6 generations to occur (Quinn *et al.*, 2001) or (iv) die. Pairing thermal tolerance data with stream (Oyinlola *et al.*, 2024) and riparian (Iacarella *et al.*, 2024) modelling can help not only examine the vulnerability of fish to ongoing warming but also address the systemic issues surrounding climate change and its effects on salmon and the aquatic environment. These projections help guide assessments and prioritization, such as Iacarella *et al.* (2024) modelling riparian growth potential and where this growth is most likely to optimize thermal stability and salmonid habitat. With this, managing populations effectively means ensuring that data are coming from relevant populations (or those experiencing similar environments), the relevant life stages present in the system are represented, seasonal differences are considered and methodology reflects goals (modelling chronic term vs acute thermal tolerance). This research adds to the growing literature demonstrating the importance of population, life cycle and methodological choice when performing thermal tolerance research.

## Conclusions

This study demonstrated differences in upper thermal limits across acclimation temperatures, methodologies and life stages for an interior population of stream-type Chinook salmon. While warmer acclimation temperature generally increased upper thermal limits, CT_max_ and CT_swim_ plateaued or declined for fish acclimated at 24°C, suggesting a ceiling for thermal plasticity beyond 20°C. CT_max_ was consistently higher than CT_swim_, and both these methods yielded higher thermal thresholds compared to the post-swim mortality results. Moreover, significant post-swim mortality occurred in both fry and parr acclimated to, and swum at 24°C, indicating an inability to recover, which seemed exacerbated in parr. This mortality was not observed in fish acclimated to cooler temperatures and swum at 24°C. This suggests that fish may be able to perform during a short-term exposure to 24°C, while prolonged exposure at this temperature is costly and not compatible with thriving. This study highlights not only the need to measure thermal tolerance in different ways but also across life stages as these may be vulnerable to elevated temperatures in different ways. For stream-type juveniles, thermal tolerance appears to vary seasonally. Small fry encounter warming temperatures throughout the summer while larger parr encounter cooling temperatures throughout late fall and winter months. Here, we found that thermal tolerance matches seasonal exposure; fry had higher thermal tolerance compared to parr, while an upper functional thermal limit of 20°C has been identified for adult Chinook salmon from interior BC populations based on intermittent flow static respirometry (Van Wert *et al.*, 2023). Given that summer temperatures in some of British Columbia’s interior streams are now routinely reaching 24°C, we would expect to see increasing levels of mortality of both adults and juveniles in future years as stream temperatures continue to warm, reaching and exceeding this temperature.

## Supplementary Material

Web_Material_coag043

## Data Availability

Data are available at Figshare: https://doi.org.10.6084/m9.figshare.32644422.
