# Supplementary material for: Effects of ecologically relevant acclimation temperature on upper thermal limits of juvenile Chinook salmon (Oncorhynchus tshawytscha)
Source: Conserv Physiol. 2026 Jul 4;14(1):coag043. doi: 10.1093/conphys/coag043 (PMC13334106; doi:10.1093/conphys/coag043)
Supplement: Web_Material_coag043 [file web_material_coag043.docx]

## Supplemental

Table S1. Summary mean ± SD, median, and mode of measured temperatures in each acclimation trough for both fry and parr life stages during the two-week acclimation period.

|  | 15°C | 18°C | 20°C | 24°C |
| --- | --- | --- | --- | --- |
| Fry | | | | |
| Mean ± SD | 14.9 ± 0.2 | 16.9 ± 0.4 | 19.3 ± 0.4 | 23.5 ± 0.4 |
| Median | 14.9 | 17.0 | 19.4 | 23.6 |
| Mode | 14.9 | 16.9 | 19.6 | 23.6 |
| Parr | | | | |
| Mean ± SD | 14.8 ± 0.3 | 16.6 ± 0.8 | 18.9 ± 0.8 | 22.6 ± 2.7 |
| Median | 14.8 | 16.6 | 19.1 | 23.6 |
| Mode | 14.8 | 17.6 | 19.0 | 23.5 |

Table S2. BIC and likelihood ratio test (LRT) for general linear model selection for CT_max_.

| Life Stage | Response Variable | Model Number | Predictor Variables | BIC | Log Likelihood | Model Selected |
| --- | --- | --- | --- | --- | --- | --- |
| Fry | CT_max_ | 1 | Acclimation temperature, mass | 176.9 | -76.2 | Model 2 |
|  |  | 2 | Acclimation temperature | 173.5 | -76.5 |  |
| Parr | CT_max_ | 1 | Acclimation temperature, mass | 59.5 | -17.5 | Model 2 |
|  |  | 2 | Acclimation temperature | 56.3 | -17.9 |  |

Table S3. BIC and likelihood ratio test (LRT) of general linear model selection for CT_swim_.

| Life Stage | Response Variable | Model Number | Predictor Variables | BIC | Log Likelihood | Model Selected |
| --- | --- | --- | --- | --- | --- | --- |
| Fry | CT_swim_ | 1 | Acclimation temperature, mass | 225.7 | -98.5 | Model 2 |
|  |  | 2 | Acclimation temperature | 221.1 | -98.6 |  |
| Parr | CT_swim_ | 1 | Acclimation temperature, mass | 401 | -186.6 | Model 2 |
|  |  | 2 | Acclimation temperature | 397.9 | -187.4 |  |

Table S4. 2-way ANOVA summary of the role of acclimation temperature (15, 18, 20^o^C) and test type (CT_max_ (stat), CT_swim_ (swim), and prolonged swim test (pro)) on mass, fork length, and condition factor for parr. Italics denote *post hoc* analysis of acclimation temperature groups (Tukey’s HSD). Note that the 24°C acclimation group was excluded from this analysis because of a mortality event and new acclimation timeline.

|  | Test type | Acclimation | | Test type * Acclimation |
| --- | --- | --- | --- | --- |
| Mass (g) | *F_(2,435)_ =* 0.718, p = 0.49 | *F_(2,435)_ =* 2.69, p = 0.069 | | *F_(4,435)_ =* 0.172, p = 0.95 |
| Fork Length (mm) | *F_(2,435)_ =* 0.673, p = 0.51 | *F_(2,435)_ =* 5.216, p = 0.0058  *20-15: p = 0.0048* | | *F_(4,435)_ =* 1.135, p = 0.34 |
| Condition factor | *F_(2,435)_ =* 7.768, p < 0.001  *swim-pro: p < 0.001* | *F_(2,435)_ =* 1.849, p = 0.16 | *F_(4,435)_ =* 0.352, p = 0.84 | |

Table S5. 2-way ANOVA summary of the role of acclimation temperature (15, 18, 20, 24°C) and test type (CT_max_ (stat), CT_swim_ (swim), and prolonged swim test (pro)) on mass, fork length, and condition factor for fry. Italics denote *post hoc* analysis of acclimation temperature groups (Tukey’s HSD).

|  | Test type | Acclimation | Test type * Acclimation |
| --- | --- | --- | --- |
| Mass (g) | *F_(2,489)_ =* 4.903, p = 0.0078 | *F_(3,489)_ =* 14.390, p < 0.001 | *F_(6,489)_ =* 14.390, p < 0.001  *swim:18-pro:15 p = 0.0038*  *swim:20-pro:15 p < 0.001*  *swim:24-pro:15 p < 0.001*  *swim:18-stat:15 p = 0.023*  *swim:20-stat:15 p < 0.001*  *swim:20-swim:15 p < 0.001*  *swim:24-swim:15 p < 0.001*  *swim:18-pro:18 p = 0.0034*  *swim:20-pro:18 p < 0.001*  *swim:24-pro:18 p < 0.001*  *swim:20-stat:18 p < 0.001*  *swim:24-stat:18 p = 0.0025*  *pro:20-swim:18 p = 0.033*  *pro:24-swim:18 p = 0.0037*  *stat:24-swim:18 p = 0.041*  *swim:24-swim:18 p < 0.001*  *swim:20-pro:20 p < 0.001*  *swim:24-pro:20 p < 0.001*  *swim:20-stat:20 p = 0.0012*  *swim:24-stat:20 p = 0.00026*  *pro:24-swim:20 p < 0.001*  *stat:24-swim:20 p < 0.001*  *swim:24-swim:20 p < 0.001*  *swim:24-pro:24 p < 0.001*  *swim:24-stat:24 p = 0.026* |
| Fork Length (mm) | *F_(2,489)_ =* 31.09, p < 0.001 | *F_(3,489)_ =* 23.13, p < 0.001 | *F_(6,489)_ =* 14.93, p < 0.001  *swim:18-pro:15 p = 0.00026*  *swim:20-pro:15 p < 0.001*  *pro:24-pro:15 p = 0.014*  *swim:24-pro:15 p < 0.001*  *swim:18-stat:15 p < 0.001*  *swim:20-stat:15 p < 0.001*  *stat:18-swim:15 p = 0.026*  *stat:20-swim:15 p = 0.0095*  *swim:20-swim:15 p < 0.001*  *pro:24-swim:15 p = 0.0014*  *stat:24-swim:15 p = 0.033*  *swim:24-swim:15 p < 0.001*  *swim:18-pro:18 p < 0.001*  *swim:20-pro:18 p < 0.001*  *swim:24-pro:18 p < 0.001*  *swim:18-stat:18 p < 0.001*  *swim:20-stat:18 p < 0.001*  *pro:20-swim:18 p < 0.001*  *stat:20-swim:18 p < 0.001*  *pro:24-swim:18 p < 0.001*  *stat:24-swim:18 p < 0.001*  *swim:20-pro:20 p < 0.001*  *swim:24-pro:20 p < 0.001*  *swim:20-stat:20 p < 0.001*  *pro:24-swim:20 p < 0.001*  *stat:24-swim:20 p < 0.001*  *swim:24-swim:20 p < 0.001*  *swim:24-pro:24 p = 0.051* |
| Condition factor | *F_(2,489)_ =* 68.69, p < 0.001 | *F_(3,489)_ =* 10.65, p < 0.001 | *F_(6,489)_ =* 10.72, p < 0.001  *stat:18-pro:15 p < 0.001*  *pro:20-pro:15 p = 0.0064*  *stat:20-pro:15 p < 0.001*  *pro:24-pro:15 p < 0.001*  *stat:24-pro:15 p < 0.001*  *swim:24-pro:15 p = 0.00091*  *stat:20-stat:15 p < 0.001*  *swim:24-stat:15 p < 0.001*  *stat:18-swim:15 p < 0.001*  *pro:20-swim:15 p = 0.03*  *stat:20-swim:15 p < 0.001*  *pro:24-swim:15 p < 0.001*  *stat:24-swim:15 p < 0.001*  *stat:18-pro:18 p < 0.001*  *pro:20-pro:18 p = 0.021*  *stat:20-pro:18 p < 0.001*  *pro:24-pro:18 p < 0.001*  *stat:24-pro:18 p < 0.001*  *swim:24-pro:18 p < 0.001*  *swim:18-stat:18 p < 0.001*  *pro:20-stat:18 p = 0.026*  *swim:20-stat:18 p < 0.001*  *swim:24-stat:18 p < 0.001*  *pro:20-swim:18 p = 0.023*  *stat:20-swim:18 p < 0.001*  *pro:24-swim:18 p < 0.001*  *stat:24-swim:18 p < 0.001*  *stat:20-pro:20 p < 0.001*  *swim:20-pro:20 p = 0.0062*  *swim:24-pro:20 p < 0.001*  *swim:20-stat:20 p < 0.001*  *pro:24-stat:20 p < 0.001*  *swim:24-stat:20 p < 0.001*  *pro:24-swim:20 p < 0.001*  *stat:24-swim:20 p < 0.001*  *swim:24-pro:24 p < 0.001*  *swim:24-stat:24 p < 0.001* |

Table S6. Critical thermal maximum at rest (CT_max_) and critical thermal maximum during swimming (CT_swim_) range and mean ± SD by life stage, test type, and acclimation temperature.

| Life stage | Test type | Acclimation temperature (℃) | Range (℃) | Mean ± SD (℃) |
| --- | --- | --- | --- | --- |
| Fry | CT_max_ | 15 | 27.9 **–** 29.5 | 28.9 ± 0.52 (n=14) |
|  |  | 18 | 28.4 **–** 30.1 | 29.7 ± 0.42 (n=15) |
|  |  | 20 | 29.5 **–** 30.7 | 30.3 ± 0.31 (n=15) |
|  |  | 24 | 24.4 **–** 30.9 | 29.1 ± 1.67 (n=15) |
|  | CT_swim_ | 15 | 27.2 **–** 28.3 | 28.1 ± 0.82 (n=30) |
|  |  | 18 | 27.5 **–** 29 | 28.6 ± 0.29 (n=30) |
|  |  | 20 | 28.5 **–** 29.2 | 28.8 ± 0.189 (n=30) |
|  |  | 24 | 24 **–** 29.4 | 28.6 ± 1.05 (n=30) |
| Parr | CT_max_ | 15 | 28.8 **–** 29.5 | 29.2 ± 0.20 (n=15) |
|  |  | 18 | 29.1 – 29.9 | 29.6 ± 0.30 (n=15) |
|  |  | 20 | 28.4 **–** 30.3 | 29.8 ± 0.47 (n=15) |
|  |  | 24 | 30 **–** 31.1 | 30.5 ± 0.31 (n=15) |
|  | CT_swim_ | 15 | 21.5 **–** 28 | 26.8 ± 1.75 (n=30) |
|  |  | 18 | 22.3 **–** 28.3 | 26.8 ± 1.52 (n=32) |
|  |  | 20 | 23.1 **–** 29.1 | 27.7 ± 1.38 (n=30) |
|  |  | 24 | 24 **–** 28.3 | 25.7 ± 1.51 (n=9) |

Table S7. Summary mean ± SD of mass (g) and fork length (mm) of mortalities < 24 hours, at 24 hours, and at 48 hours.

| Life Stage | Body metric | Mortalities under 24h | Mortalities 24h | Mortalities 48h |
| --- | --- | --- | --- | --- |
| Fry | Mass (g) | 0.64 ± 0.085 (n=2) | 0.54 ± 0.075 (n=4) | 0.62 ± 0.11 (n=7) |
|  | Fork Length (mm) | 39.5 ± 0.5 | 36.2 ± 1.43 | 36.9 ± 0.99 |
| Parr | Mass (g) | 6.39 ± 0.56 (n=20) | 5.77 ±0.18 (n=25) | 4.98 ± 0.48 (n=14) |
|  | Fork Length (mm) | 85.4 ±1.65 | 83.0 ±1.11 | 76.0 ± 1.59 |
